# Investigating the healthcare pathway through patients’ experience and profiles: implications for breast cancer healthcare providers

**DOI:** 10.1186/s12913-020-05569-9

**Published:** 2020-08-11

**Authors:** Emna Cherif, Elisabeth Martin-Verdier, Corinne Rochette

**Affiliations:** 1 IAE Clermont Auvergne School of Management, CleRMa, University Clermont Auvergne, F-63000 Clermont–Ferrand, France; 2 Founder and holder of the Research Chair Health and Territories, University Clermont Auvergne, Clermont–Ferrand, France

**Keywords:** Patient experience, Healthcare pathway, Touch points, Patient profiles, Breast cancer, Netnography, Qualitative study, Online communities, Emotional support, Informational support

## Abstract

**Background:**

Healthcare systems are facing many changes. Particularly, patients are more engaged in the care process. The medical perspective of the process is insufficient to provide patients with high quality care and service personalisation. This research presents an attempt to complete this medical perspective through an experiential perspective, especially for chronic diseases such as cancer. We investigated patients’ experiences and profiles to reach a deeper understanding of their needs and expectations when they confront the disease.

The objectives of this research were to model the key stages underling the patient pathway and to identify the challenging touch points of the interactions between patients and healthcare providers. Bringing together findings of patient experience, pathway, and profiles would help all the stakeholders involved to develop better practices for the healthcare process.

**Methods:**

A qualitative observational nethnography on a French specialized forum for breast cancer patients “*les Impatientes”* was conducted. A total of 967 reviews were collected over a complete year period from all over France. Thematic and lexicometric content analysis were performed according to the experience dimensions, the pathway stages and touch points, as well as the patients’ profiles.

**Results:**

Data analysis shows that the healthcare pathway experienced by the patients is built around three stages. The discovery stage is closely related to the emotional dimension regarding the patient and physician relationship. The examination stage is characterized by a more technical and informational needs for the types of treatments. The follow-up and survivorship stage illustrates the patients’ need to assess the treatments’ effectiveness and the quality of the follow-up. Moreover, three profiles of patients were identified. The newcomers, the altruists and the autonomous are characterized by different attitudes depending on the stage of the healthcare pathway they were living.

**Conclusions:**

Our research presents an original modelling of the patient pathway and profiles beyond the medical process. It gives practical tracks to improve the healthcare pathway. Patients expect healthcare providers to integrate and strengthen several challenging touch points in order to create satisfactory patient experiences and high quality service.

## Background

Healthcare organizations are facing many economic challenges. In the one hand, increasing healthcare costs threaten their survival and care delivery quality (e.g. national health expenditures reached € 203.5 billion in France in 2018^1^ and £197.4 billion in the United Kingdom in 2017^2^). On the other hand, patients are paying rising costs for healthcare services (e.g. In France, they spent up to € 3037 a year out-of-pocket in 2019^3^ and £2989 in the United Kingdom^2^) and are more demanding regarding the quality of their care and the service personalization [1–3]. Patients no longer interact with the healthcare team as passive recipients of services [4, 5]. They get involved in collaborative interactions all along the care process to improve their healthcare experience, especially for chronic diseases such as cancer [6, 7].

Although the role of the patient in the healthcare process is gaining a growing interest, little research has focused on the patient healthcare experience [8, 9]. Experiential theorists consider experience as a multidimensional construct that includes cognitive, emotional, social, sensorial and behavioural responses in any direct or indirect interaction with the organization [10–12]. Particularly, experience consists of an iterative and dynamic pathway that covers every interactional offline and online touch point [13–17].

Existing studies have mainly focused on the healthcare pathway through a medical perspective. The HPST law^4^ defines the care pathway through a temporal (effective sequencing of care delivery) and a spatial dimensions (care delivery in or near the patient’s home and region). Prior research investigated the right orchestration of consultations and care delivery [7, 18–21], as well as the role of rigorous teamwork design [22, 23] as key factors to improve healthcare process and patient experience.

However, such medical perspective of the process provides an incomplete understanding of patients’ needs and expectations during the whole care experience. Further, it brings a limited identification of the troubles experienced by patients, which must constitute points of attention for healthcare providers to implement better practices. Through this study, we try to fill this gap. Drawing on previous work on customer experience and pathway [14–16, 24–26], we aim to show how patients experience their healthcare pathway over time and through the various stages of the treatments. This study aims to complement the scarcity of studies on the topic. It provides a practical modelling of the patient pathway with challenging touch points for all the stakeholders involved in the process. It may help providers implement appropriate and responsive programs that better fulfil patients’ needs.

Furthermore, patients differ in their needs, beliefs and involvement along the healthcare process [27–31]. These differences influence patients’ experience through the multiple touch points at each stage of the healthcare pathway. This study aims to reach a deeper understanding of patient disparities along the healthcare experience to put forward a patient typology. Our study focuses on a particular category of patient and illness: patient touched by breast cancer and seeking social support on online communities [32, 33]. Although customers’ typologies have been studied for a long time [34–36], there is insufficient research investigating patient typology based on how patients perceived, use healthcare services and experience their healthcare pathway. Therefore, research that delves deeper in this field can supply healthcare providers with a set of specific practices according to the patient profile. Moreover, from a practical point of view, it is of great interest to integrate together the patient pathway and the patient profile to give a better understanding of their contribution to improving the experience of the healthcare delivery.

A qualitative observational nethnography was conducted to address the research issues. Nethnography is an adjustment of the ethnography method to online communities’ characteristics. Thus, it is a qualitative research methodology for using, collecting, analyzing and interpreting the information publicly available online. Compared with traditional qualitative methodologies, netnography is less time-consuming, simpler, and less costly than market-oriented ethnography. It is also more naturalistic than focus groups or personal interviews and entirely unobtrusive [37, 38].

The study’s focus is on breast cancer patients as it covers a long healthcare process and represents opportunities to investigate a complete patient pathway. In addition, cancer patients are particularly involved in their healthcare experience as the healthcare process is still complex and poorly coordinated [6, 7, 39, 40].

## Methods

### Data collection

We focused on breast cancer patients, who had received, are still receiving or are scheduled to receive a cancer treatment. Cancer covers a long healthcare process and represents opportunities to investigate a complete patient pathway. Moreover, cancer patients are especially engaged in the healthcare experience [6, 7, 39, 40] .We conducted an “observational nethnography” [37, 38] on a French specialized forum for breast cancer patients: *les Impatientes*. This forum is a reference for all patients who look for support, or want to learn and gain feedback from other patients with similar experiences. Previous studies suggest that social support is very helpful and beneficial for patients suffering from serious diseases [41] Thus, oncologists advise this forum to their patients although it is not moderated by a medical team to verify the veracity of the shared information [42]. The choice of this online support community was motivated by the sensitivity of cancer patients, who may be emotionally susceptible to this approach than to other methods such as interviews or questionnaires. Indeed, the anonymity guaranteed by the forum makes it possible to comfort and give confidentiality to participants without the interference of biases related to a third person. Moreover, as suggested by [43], we did not disclose our presence to the community to avoid inhibiting members’ discussions. The forum provides an open-access for patients to ask their questions in order to get answers and interactions with other members at any time and from all over France. It contains four distinct sections - “I’m new, practical help, patient-doctor relationship, and survivorship and follow-up”- to help patients to ask their questions in the right one. Moreover, as it was hard to track the whole pathway for each patient, we relied on these sections as a timeline for patients’ comments to point out their experiences through the stages of the pathway.

We have chosen to analyse the reviews published over a complete year period (from July 2016 to July 2017). The data was time-consuming to gather and analyse as all the steps were done manually from collection to interpretation. Collected data provides very rich primary source information about their pathway from the discovery of the disease to follow-up and the survivorship stage.

In order to model the patients’ healthcare pathway along the various stages of treatments, we focused particularly on the types of concerns experienced by patients. Prior studies have come to agree that experience involves a multidimensional interaction through sensorial (senses and how they arouse pleasure, excitement, satisfaction, etc.), affective (moods, feelings, and emotions), cognitive (thinking and conscious mental processes), physical (practical act of doing something and usability), and social (relationships, that occur during common experience) touch points [15, 26, 44]. Furthermore, recent studies on patient-breast cancer medical pathway highlight two major dimensions – the temporal dimension refers especially to the effective sequencing of the care delivery and treatments, and the spatial dimension related to the proximity of healthcare centres [18–21]. Thus, we consider that each concern refers to an experience dimension and might constitute challenging touch point for healthcare providers.

In addition, patients’ typology identification was based on a set of characteristics^5^ we used to assign patients to homogeneous groups [36, 45]. Thus, we simultaneously analysed observable characteristics through the stages of the pathway, unobservable characteristics through the perceptions and the lived experiences, and situation-specific variables through patients role - givers and/or seekers- of informational and/or emotional support on the forum [44, 45] to identify the patients profiles.

### Data analysis

We conducted a thematic content analysis on a total number of 967 comments collected (121,097 words). Authors carried out independently the iterative reading process and thematic identification [46]. The coding of the data followed the three main stages of the pathway and the challenging touch points [47]. The content analysis also focused on patients’ typology identification. The profiles were identified post-hoc as the number of segments derives from data analyses. Following [36], it consists on a descriptive typology, as no interdependence among bases exists. In order to refine our analysis and reach in-depth understanding of the reviews, we also applied a lexicometric analysis to the data using the Tropes® software [48, 49]. Tropes® software enables hierarchical classifications based on a semantic rapprochement in the meaning of words.

## Results

### Experience dimensions

Results from lexicometric analysis provide an overall view of the corpus and enable the identification of the experience’s most relevant dimensions. The data highlights the importance of the temporal dimension with 2872 semantically equivalent words such as “time”, “weeks”, “months”, “years”. Patients also use precise dates to describe the events (“on September 5^th^”). This is consistent with the nature of the disease that requires care and treatment over time. In contrast, the spatial dimension emerges less from the corpus with only 187 semantically equivalent words (place, region, next to). The spatial dimension includes two sub-dimensions: the geographical (next to me) and the symbolic (my horizon darkens …). The emotional dimension is the third category revealed by data (1254 of semantically equivalent words). It covers positive words (trust, hope, smile, happiness), but also negative words (sadness, anxiety, panic, fear, worries, fears, grief, pain, shame, humiliation, anxiety, fear, etc.). Social and cognitive dimensions of experience were also highly expressed by patients. For the social dimension, words refer especially to the “family” (394 of semantically equivalent words) and “relatives” (119 of semantically equivalent words). The cognitive dimension is recognizable with words such as “know” (357), “understand” (148) and “ask” (107). The behavioural dimension refers to the action with the use of verbs such as “do” (744), “find” (168), and “try” (80).

These dimensions are present all over the patient pathway through each touch point.

### Patient pathway

One on the main issues of the research is to identify the healthcare process: the stages followed over time and the environment (physical and virtual) frequented by the patients before, during and after treatment(s). Patient pathway is not only addressed from a medical perspective but also throughout the care pathway experienced by patients when living the situation and confronting the disease. The content analysis shows three main stages:

#### The discovery stage

The announcement of the disease is closely related to the emotional dimension of the experience. Patients use words and terms like “shock” (49), “blow of the club” (2), “tsunami” (4). Also, they perceive the announcement generally as “brutal” (3), “terrible” (12) and “a source of anguish” (24), “anxiety” (6), “chaos” (1) and “fear” (195).

**Fig. 1:**
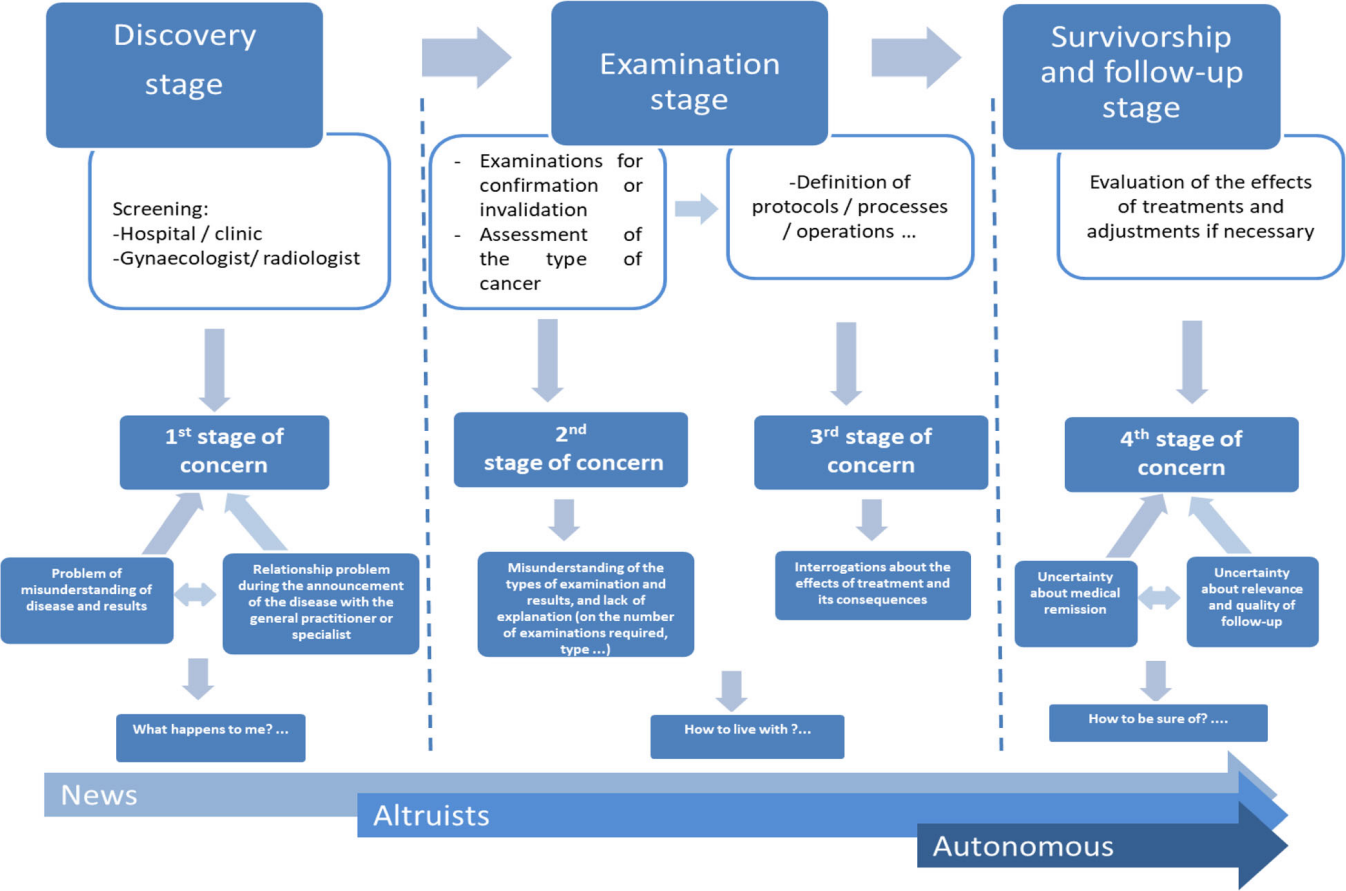
Modelling the experience of the patient pathway

Results from the thematic analysis show that the discovery stage is a particularly sensitive phase regarding the patient / physician relationship. This stage constitutes a first phase of anxiety (see Fig. 1). The misunderstanding of the disease on one hand and the relational difficulties with the doctors on the other hand are sensitive and challenging points of attention:

“On September 5th a biopsy, on the 10th the lab sends the results to the radiologist, the 17th having no news, I call the office of the radiologist, the secretary tells me there was a problem with the mail, in fact they had not sent anything. I call my General Practitioner, he asks for a copy of the report at the lab …. The next day I receive the radiologist's mail which puts “doubtful cells” and “I insist that you contact your doctor”. I remind my doctor, it's September 18 and he says it's a cancer. Great phone call! It's Friday. The weekend will be great. On Monday he told me just carcinoma no more details”;

“I see a pit-bull comes out that bears the name of a radiologist who shouts at me saying that a breast like that was not normal etc. etc. He tells me: madam, you have breast cancer, the sky falls on my head. ”;

“It’s like a nightmare since Tuesday”.

At this stage, all the patients adopt the same attitude of shock and misunderstanding. The announcement of the disease is followed by an intensive request for information about the different treatments, the meaning of the medical terms and the cure rates. Patients use online discussion forums to find the information they lack. Online communities also help them to supplement the explanations provided by the doctors, usually perceived as insufficient. They want to feel supported and especially reassured by patients who have experienced similar experiences:

“I feel like I'm on a path where many women may be walking at different stages but I'm not the only one on the road.”;

“If I can have opinions of people in the same case as me.”

At this stage, we identified two types of touch points that should be of great interest to the healthcare service providers: informational touch points and emotional touch points.

#### The examination stage

This stage is characterized by the word “examination” (125). Patients feel a strong anxiety about the types of examination and the results, as well as the treatments and their effects. The analysis reveals more technical content on:

- the nature of the examinations and the interpretation of the results:

“Anatomic-pathology, also called pathological anatomy, is the medical discipline forming part of the study of diseases. It studies the lesions and structural changes of organs and tissues, caused by a disease. ”;

“Your tumour is less than 2cm, it is operable immediately, pet-scan is ok and no lymphadenopathy echo”.

“Bcc: infiltrating ductal carcinoma (3 3 2 = high grade), and proliferative marker (ki 67 to 80% = very high rate of the mitotic index). The very high ki67 means that the tumour is very aggressive. As for the “sarco … ” contingent, normally unknown to the “battalion” of the tumour cells of the breast, I think that these are the cells that are very undifferentiated (the normal cells of the breast are differentiated) Triple-negative breast cancer”.

- the treatments and their effects:

“It may be your Ki67 that explains the chemo (“E.C. and 3 Taxotere) compared to mine (3 F.E.C. and 3 Taxotere); ” Nausea insomnia and morale at half-mast for 4 days after the 4th injection of granocytes. The oncologist prescribed 7 injections as of d + 6 ”;

“The oral chemo protocol is the following: Endoxan 50 mg tablet 1tbd^6^ / day + methotrexate 2.5 mg 1 tbd morning - Monday, Thursday 1 tbd morning and evening + herceptin IV I started on October 14, 2015 and I confess that it is hmm hmm not top. Side effects: headache (==> CEREBRAL MRI), pain in jaw and teeth, sinus in the face, fatigue ++++, joint pain everywhere (cervical, shoulder, back, pelvis, elbow, wrists …) and nausea ++++”.

Patients also seek evidence to trust the healthcare providers involved in their care (“Your priority is to choose the right hospital and the right caregivers.”)

At this stage, patients express their needs particularly for informational touch points.

#### The follow-up and survivorship stage

Lexicometric analysis shows a dominance for the word “check-up” (102) associated with medical examinations such as: “mammography”, “radiography”, “ultrasound”, “assessment”, “The anxiety of the biannual check-up after breast cancer”, “from one centre to another the check-up protocols are very variable”, “one really has the impression of a two-speed follow-up, “from one centre to the other the follow-up is sometimes the minimum union”, “I for the follow-up level I confess that I do not understand everything”.

Thematic analysis demonstrates the need for patients to assess the quality of the follow-up and the importance of the performed tests just after the treatments:

“At the end of the treatment, I had no radiological examination to confirm that the treatment had worked or not. Then I did it out of my own pocket. I asked my onco gyneacologist to give me a prescription .... at my expense but at least I was reassured. The results were good. My oncologist was surely right to wait 6 months but one more exam is good for morale … ”;

“I had my appointment with the oncologist and I asked him the question of follow-up after treatments ... and he confirmed that mammo + echo check-up after 6 months after stopping the rays. “It is long”, I told him and he told me that it was the protocols ... I told him that I would never be able to wait for 6 months, to resume my activity without knowing if all the treatments were effective ... and he said “we will see at that time! ” Since I will be under Herceptin for one year (HER +++), he told me that I was covered (except that I have read cases where even some Herceptin cases had relapses or metastases). I will see with my surgeon or General Practitioner to see what can be done if he does not want to give way! ”;

“Most projects are about the “how to live with”. But, I am interested in knowing the percentage of recurrences in tamography on a percentages of overall recidivism ... in fact X% of patients recidivate but 13% of these X%: that gives what percentage on arrival? And what quality of life...”.

At this stage, patients need guarantees about treatment effectiveness and remission. Informational touch points should constitute support touch points for the healthcare institutions and the development of a management that would be better perceived by the patients.

Content analysis enabled identification of three patient profiles in the experienced healthcare pathway.

### Patients typology

**Fig. 2:**
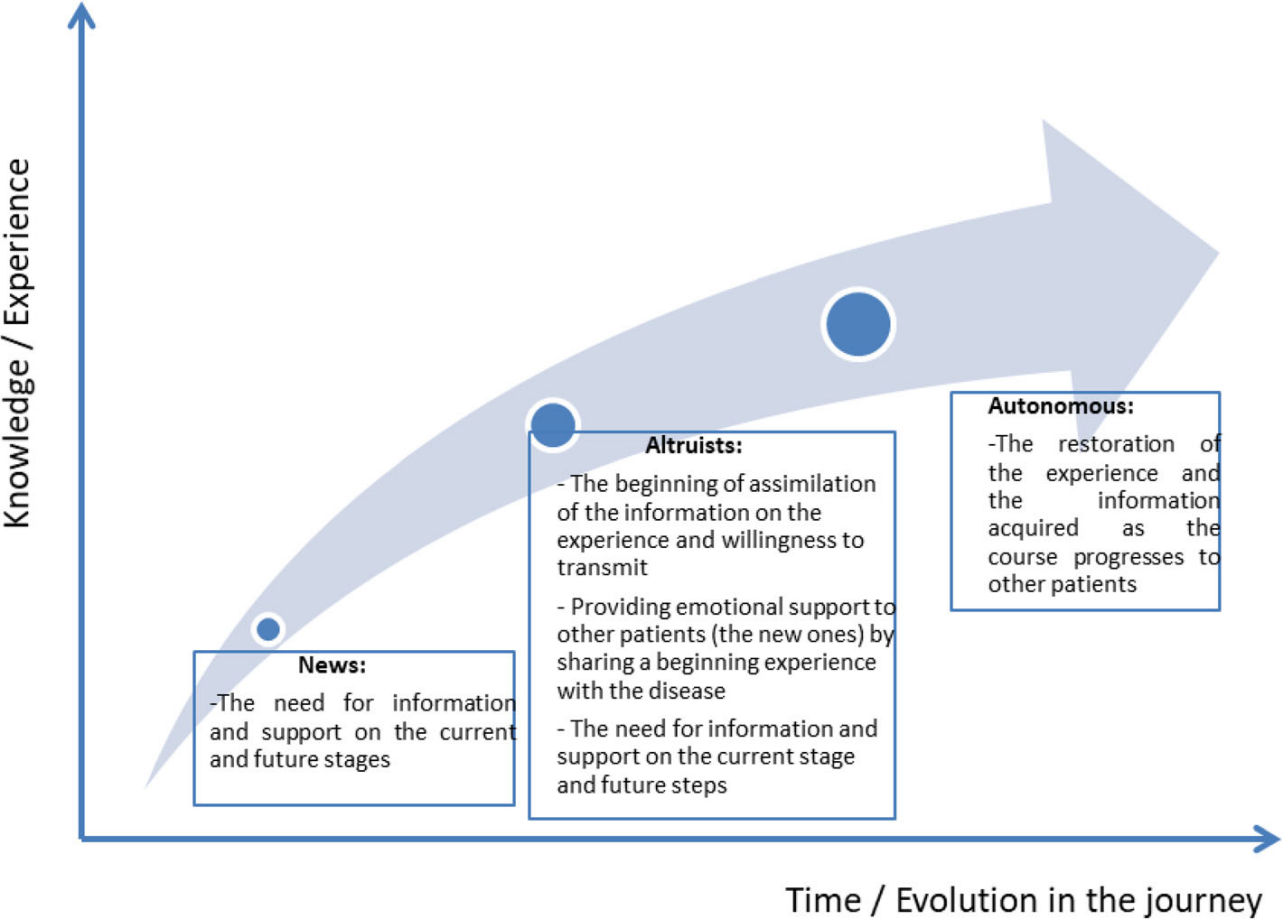
Patients typology and experiential pathway

Both observable and unobservable characteristics of the patients are considered in the identification of the profiles [36]. The simultaneous study of the observable characteristics, through the stages of the pathway, and unobservable, through the perceptions and the lived experiences, allowed us to identify three profiles of patients (see Fig. 2).

*The newcomers* are mainly in a phase of information search. They try to fill the gap in information and / or a lack of understanding of the information that has been given to them. They rely on the doctors but especially on the other patients to inform them (“I lack information....Who can explain if he knows how to have info on the subject.”; “are there women in the 33 who would like to get in touch to exchange “).

Figure 2 shows that patients may be new throughout their experience of the disease. At each new stage of the pathway, they are faced with new concerns often related to misunderstandings or lack of information. In the discovery stage, they have just learned the verdict and seek support from other patients. The emotional dimension of the experience is clearly seen through verbatim as “I’m lost ..... I’m trying to stay strong for the kids. “ or “ I’m in shock. “.

A strong cognitive dimension also marks this step. Patients generally have very vague notions of their case and are not able to undertake research on their own (“So I’m looking for people like me who had non-invasive cancer in situ and who had a bilateral mastectomy with immediate reconstruction.”).

During the examination and treatment stage, patients are still lacking information. They are new compared to the knowledge of what the exams cover and their results as well as the effects of the treatments followed (“acronyms that I do not know “petscan”^7^ what is compared to scan? Already had an injectable scan. It has a relationship or not? What is a FEC? “).

Finally, in the follow-up and survivorship phase, patients will question the quality of the medical follow-up and the examinations (“I have to check every 3 months, my oncologist has retired, I saw him for the last time in December. Another oncologist had to take over, it is a new one I do not know. What it was surprising when I wanted to take an appointment for the end of March, they answered, and not very kindly, that there was no place until July. I explained that I could not wait 7 months but apparently my case does not seem to interest them. “).

The cognitive dimension prevails over the last two stages of the patients’ pathway.

*The altruists* wish, above all, to share the beginning of their experience of the disease on the informational level as well as on the emotional and social levels. Altruists understand a lot of information about the stages of the pathway that they have already faced (especially the discovery stage). They want to share this with the other patients; especially with newcomers (“I invite you to look at my bio. I have noted a lot of information that will help you, hopefully!”).

The cognitive dimension of the altruists swings between information transmission and information requesting. Patients are still asking for information about the coming stages of the pathway. They need to learn about examinations and the treatments stage or follow-up and survivorship stage (“I intend to push the oncologist to his limits to see what he proposes ... but I need TAP Scan: View of the thorax, abdomen, organs. TEP Scan: better than the previous one but does not allow to see the bones or brain - Cerebral MRI: view of the brain only (I was refused during my extension report) - bone scanning: bone scan only. The ideal would be to do a Pet Scan, bone scintigraphy and MRI cerebral so if we want to check everything? ?? ”).

Altruists are still in need of emotional support. They have doubts and need assurance and reassurance regarding the progress of their care. They try to discuss with patients who are able to understand the tests they are passing through (“Small passage between my series of medical examinations for my first follow-up check after Chemotherapy and radiations..., currently under hormone therapy (tamography and enantone) ... Side echo and mammo were OK: Side small nodule in the belly (in the area of enantone injection): granulomatous fibrous nodule, centimeter, hyper vascularized located in the sub-dermal region para-umbilical likely in relation to the recent injection. Normal and continuous appearance of the cutaneous and muscular planes, no collector’s image, no zone of disorganization. Cardiac echo OK... Finally, I hope.. ”).

At the same time, the altruists gain empathic feeling, especially for the newcomers. They share their experiences in order to reassure, accompany them in their anxieties and fears and give them the emotional support they need (“I think of you Celine, like all of us, who no longer read you I worried for you and hope you are well.... It’s up to us to support you I cross my fingers for you so that this chemo is effective you have to believe it YOU’LL GO UP THE SLOPE”).

The emotional and social dimensions are therefore interwoven. It should be noted that this altruistic profile emerges from the second stage of the pathway and extends to the last stage (Fig. 2).

*The autonomous* act as a guide and an expert. The autonomous patient represents a stable and informed figure among the community. They are usually patients who have been diagnosed for some time and have more experience and are more informed than others (“You can write to us if you want us to help you find a competent institution knowing that to have access to anti-cancer centres, you must have a questionable mammogram”). Patients show an ability to become autonomous from the third stage of the pathway. They interact on the forum to share their experiences and the information learned as the process progresses (“I also had an ablation and I will go to the reconstruction by diep (no other possibilities). A friend who has done her reconstruction by prosthesis has to change it about every ten years. She has a hard time getting shell at first. Now it is also a matter of person I prefer to use components of my body if it is possible … rather than a foreign body less natural to the aspect also …. which does not flatten in position. Other members will certainly give you their opinions …”).

The autonomous respond to the questions of all the patients and share a great deal of information, whether medical or personal, to help them in their pathway and to accompany them in times of distress (“Breast screening by Mammography, followed by an ultrasound, remains the reference in the prevention of breast cancer and its early detection is essential … The risk of radiation-induced cancer is very small compared to the benefit of the screening. Of radioactivity, but if it detects the tumour masses, it does not detect micro calcifications in contrast to mammography.”). Their approach is more factual and rational where the cognitive and social dimensions of the experience dominate.

The identification of the three patient profiles allowed us to model them from a temporal (stages of the pathway) and experiential (accumulation of knowledge) perspective. Figure 2 shows on the abscissa the evolution of the profile over time and on the ordinate the lived experience. Over time and through the various stages of pathway, patients gain experience of the disease. They are more knowledgeable and want to share information more intensively. They develop a certain “expertise” and a more reasoned attitude in the transmission of the information and their knowledge.

Moreover, the dimensions of the experience differ from one profile to another. The newcomers need above all information and have little experience of the stages of the pathway in which they find themselves. They are not exclusive to the disease discovery stage but may also be considered new during the examination stage and the follow-up and survivorship stage. Even if the cognitive dimension from an information acquisition perspective prevails strongly in their behaviour, the emotional dimension is also very present.

Altruists have assimilated some of the information they can share (cognitive dimension from a perspective of transmission) and they have a more or less important experience in the care pathway. The dimensions that prevail for them are emotional (emotions partly controlled) and social (empathy, support). Nevertheless, they remain in an active process of information acquisition.

Finally, the autonomous are characterized by a rationalization of the lived experience and a willingness to share (social dimension). The cognitive dimension (willingness to transmit what they know) is also very strong.

The behavioural dimension that underlies the three experiential dimensions previously mentioned is present in these profiles. It manifests in all the patients by decision-making such as changing the follow-up structures, the demand for prescriptions for complementary examinations (“I said goodbye to my Parisian hospital after 25 years, today ‘I am ready to change oncologist for better cancer monitoring’, ‘I have a PET scan (at my request with my oncologist)’ ”).

## Discussion

### Key findings

This research confirms the existence of three main stages in the healthcare pathway: a discovery stage, an examination stage, and a follow-up and survivorship stage. These results converge with previous work on the customer’s pathway, which is structured around three key stages: the prepurchase, the purchase and the postpurchase stage [17, 24–26]. At each stage, the analysis highlights the badly experienced moments during the pathway. Especially, results revealed relational and informational concerns throughout the pathway. Namely, the medical protocols (examination, treatments, etc.) are similar in both public hospitals and private centres. This is line with previous findings regarding the temporal and spatial dimensions of the health care process [7, 18–21]. However, patients experienced the process very differently from an experiential perspective. The temporal dimension is rather associated with significant milestones that punctuate patients’ lives. The spatial dimension, beyond its purely geographic scope, is experienced in a more symbolic way by the patients, as their own world became sad and painful. Furthermore, results show that critical touch points followed two main dimensions: the informational (cognitive) dimension and the relational dimension (emotional and social) [32] Most patients initially seek support that they do not always find in the medical and nursing team. They need to feel surrounded and supported by people who understand their anxieties and distress. Patients deplore a significant information deficit and highlight problems of misunderstanding at all stages of the pathway, partly due to poor dialogue and insufficient listening. They will therefore try to find the information outside of the medical team. Thus, the medical dimension (professionalism, expertise) is only one component among others in determining the patient pathway and in particular those related to cognitive (request and transmission of information) and relational (empathy, support) dimensions of the experience.

We may note that patients do refer to the nursing staff in their comments. However, the latter can play a key role in building a positive patient experience, particularly in complex care pathways [50]. For instance, some countries (e.g. USA, Canada) have developed new nursing functions, such as case managers and nurse navigators, to support patient navigation [51]. These new supportive and informative roles generally based on nursing functions are still not very widespread in France. The first initiatives date back to 2010, but they are gradually spreading and constitute one of the main lines of the “my health 2022” program^8^ presented by President Macron.

### Contributions

Through this study, we highlight a number of contributions. First, our research emphasizes the key role of touch points in building patient experience. It confirms the existence of a patient pathway, which restores the experience and expectations of patients in a more complete way than the medical process. Healthcare providers should pay particular attention to these critical touch points in order to improve the satisfaction of patients during the healthcare pathway and increase service quality. Our recommendations go in this direction and are formulated according to the categorization of touch points suggested by [17]:

- The first category of touch points of the customer experience (here, the patient) is the brand-owned touch points. Here, hospitals, cancer centres, healthcare service providers constitute brands. They generally carry a positive perception associated with their expertise. Some healthcare organizations, on the other hand, lose the good reputation of the institutional brand because they are perceived as “dehumanized medical factories”. The “human” aspect of the centres and hospitals is as important to patients as the treatments they follow. Indeed, many patients are considering changing hospitals, centres or oncologists if they feel neglected by the medical team. Patients seek the empathy and the availability of the medical team. Establishing a charter of commitments around attention, the ability of doctors and staff to take the time to listen and respond to patients’ concerns could be a good practice. A referent to follow-up the patients along the stages of their pathway in the image of the “case managers” should also be considered. We emphasize the need to train and dedicate staff to the management of the patient pathway so that they feel better accompanied. Trained staff could also facilitate the coordination between all the actors: physicians, oncologists, and radiologists. The Gustave Roussy Institute project set out this recommendation: “to improve the management of the pathway by developing new functions (such as case managers) designed to improve every aspect of the healthcare process of the patients, their experience in all its dimensions and at every stage of the disease, but also to provide useful and innovative services such as the website and the mobile application offering a personal digital space “MonGustaveRoussy”.
- The second category concerns the touch points associated with the partners - those designated and / or over which the organisation has some control. There is still much work to be done in terms of identification and coordination with these partners (cancer check-up centre, therapeutic education associations). This work would allow a better orientation of the patient and would contribute more strongly to generate a positive experience. The creation of networks of coordinated health actors such as cancer networks must be a priority for hospitals.
- The third category of touch points relating to the patient itself is quite ambiguous. We can hypothesize that the absence or near absence (this would need to be confirmed by future research) of these points of contact is a strong feature of the nature of the disease. Oncological treatments follow rigorous protocols that do not leave many initiatives to patients to act by themselves. Nonetheless, healthcare providers should focus on the follow-up stage, where some patients are unable to wait the 6-months waiting period recommended in the healthcare protocol between the end of treatment and the follow-up examination. They, therefore, decide to carry out the examination tests by themselves just at the end of the treatments.
- Finally, the fourth category covers the social and external touch points, that provide a very positive overall experience. Indeed, the influence of other patients and peers is major in its influence on the perception of care pathway. Other patients constitute a real resource to activate in a framed way. The sharing of experience, both with other patients and the medical team, appears to be a particularly important marker for those patients experiencing an emotionally strong and painful experience. This research emphasises, for breast cancer, a lack of shared experience with the medical team.

Furthermore, our study provides an original approach to patient segmentation. Generally, patient profiles are built on clinical criteria. We propose in this study that patient profile is built on behavioural criteria and attitude towards the social network - - givers and/or seekers of informational and/or emotional support [32]. This approach provides another useful approach to patient profiles to better understand the health care experience.

### Limitations and future research

Our study focused only on breast cancer patients involved in online support communities. They have a certain degree of autonomy to understand and cope with the disease - they seek informational and emotional support and they express the desire to share things related to the disease. Passive patients are therefore not included in this study. A second step to complete the study of experiences and pathways requires a different data collection methodology for patients who completely defer to the decisions of the medical team and allow themselves to be guided through the medical process only. One of the authors is currently working on this category of “submissive patient” or “dependent patient”, as one of its characteristics is the low degree or absence of patient autonomy.

Future research could also therefore extend the results to other types of pathology in order to increase the validity of the results. The profiles and stages identified in this study concern women and one type of cancer: breast cancer. It has specificities compared to others: high prevalence, systematic national screening, a 5-year survival rate of 75–80%, a treatment that combines several therapeutic modalities (chemotherapy, radiotherapy, hormone therapy, and targeted treatment). National public health action in favour of systematic screening for women from 50 to 75 years old probably leads to greater awareness in this population. Moreover, in terms of health, women have less risky behaviours than men (smoking, alcoholism) [52], which could also explain some of the reactions when the diagnosis is announced for those following a healthy lifestyle. Overall, women have a better knowledge of the health system. Thus, gender is not neutral on the way in which the patient experience is constructed [53, 54]. The gender issue has only very recently been included in studies in France on attitudes towards health and the way they influence the care pathway [55]. Therefore, replicating this study on other types of cancer (without prevention, low prevalence, urgency related to the rapid progression) and on a male population will make it possible to identify constants or specificities in the pathway and the way they experience it. The extension of this research will make it possible to confirm (or not) whether the patient typology presented in this study is identifiable for other cancers and for male patients, or if it is possible to identify others. Similarly, it would be relevant to identify the behaviour and attitude of patients during cancer recurrence or the development of another cancer than the one experienced by also integrating the question of age referring to works underlining the stoic attitude of older patients towards the disease [56].

## Conclusion

Healthcare providers have long focused their attention on the medical care process of the patient pathway. This study provides a first attempt to complete this medical perspective through patients’ experience and profiles. Based on 967 reviews of patients with breast cancer, we model the patients’ experience in a three main stages pathway: the discovery stage, the examination stage, and the survivorship and follow-up stage. Healthcare providers need to pay particular attention to the relational and informational dimensions of the experience according to the stage of the healthcare process and patients’ profiles. The pathway modelling through the several challenging touch points highlighted in this study is a starting action for all the stakeholders involved in the healthcare process.

## Data Availability

Dataset files are available upon request from corresponding author.

## Abbreviation

HPST law is the French law “Hôpital Patient Santé Territoire”: Hospital Patient Health Territory for reforming the hospital

## Footnotes

1 https://drees.solidarites-sante.gouv.fr/IMG/pdf/infographie-cns2019.pdf

2 https://www.ons.gov.uk/peoplepopulationandcommunity/healthandsocialcare/healthcaresystem/bulletins/ukhealthaccounts/2017

3 https://www.lefigaro.fr/conjoncture/les-depenses-de-sante-ont-atteint-3037-euros-par-francais-en-2018-20190910

4 French law n° 2009–879_ 21 July 2009 reforming the hospital and relating to patients, health and territories, JORF N°0167 2009;12,184.

5 Wedel and Kamakura (2000) argued that the market segmentation is based on a “set of variables or characteristics used to assign customers to homogeneous groups”. The profile of individuals in each group may rely on observable (e.g. hospital choice), unobservable (e.g. satisfaction, behaviors or attitudes), general (e.g. demographics, lifestyles) or situation-specific characteristics (e.g. product/service choice and usage [36, 45].

6 tbd = tablet per day, tb is the abbreviation for drug tablet

7 Petscan is the abbreviation of Positron Emission Tomography Scan

8 https://solidarites-sante.gouv.fr/IMG/pdf/ma_sante_2022_pages_vdef_.pdf
